# Survey on Self-Supervised Learning: Auxiliary Pretext Tasks and Contrastive Learning Methods in Imaging

**DOI:** 10.3390/e24040551

**Published:** 2022-04-14

**Authors:** Saleh Albelwi

**Affiliations:** 1 Faculty of Computing and Information Technology, University of Tabuk, Tabuk 47731, Saudi Arabia; sbalawi@ut.edu.sa; 2 Industrial Innovation and Robotic Center (IIRC), University of Tabuk, Tabuk 47731, Saudi Arabia

**Keywords:** self-supervised learning (SSL), auxiliary pretext tasks, contrastive learning, pretext tasks, data augmentation, contrastive loss, encoder, downstream tasks

## Abstract

Although deep learning algorithms have achieved significant progress in a variety of domains, they require costly annotations on huge datasets. Self-supervised learning (SSL) using unlabeled data has emerged as an alternative, as it eliminates manual annotation. To do this, SSL constructs feature representations using pretext tasks that operate without manual annotation, which allows models trained in these tasks to extract useful latent representations that later improve downstream tasks such as object classification and detection. The early methods of SSL are based on auxiliary pretext tasks as a way to learn representations using pseudo-labels, or labels that were created automatically based on the dataset’s attributes. Furthermore, contrastive learning has also performed well in learning representations via SSL. To succeed, it pushes positive samples closer together, and negative ones further apart, in the latent space. This paper provides a comprehensive literature review of the top-performing SSL methods using auxiliary pretext and contrastive learning techniques. It details the motivation for this research, a general pipeline of SSL, the terminologies of the field, and provides an examination of pretext tasks and self-supervised methods. It also examines how self-supervised methods compare to supervised ones, and then discusses both further considerations and ongoing challenges faced by SSL.

## 1. Introduction

Deep learning algorithms have obtained state-of-the-art performance in broad applications of computer vision, such as image classification [1,2], object detection [3], and image segmentation [4,5]. These methods have succeeded due to the prevalence of large-scale, readily available datasets with manual annotations. However, collecting these input–output pairs for training is expensive, time-consuming, and labor-intensive. In some domains, such as the medical field, collecting data is even more difficult because appropriate datasets are limited or unavailable. Even when these datasets exist, they often require annotations, the addition of which requires knowledge of the medical field and can be time-intensive. Supervised learning is also susceptible to generalization errors, adversarial attacks, and spurious correlations, which all cause additional bottlenecks [6].

Because of this, most current research focuses on adaptable systems that account for new conditions without requiring extensive supervision. This has led to advances in subfields such as transfer learning; semi-supervised, weakly supervised, and unsupervised learning; and self-supervised learning [7].

Transfer learning [8] is a popular approach to counteract the lack of annotated datasets. This technique uses research, which focuses on the storage and application of problem-solving information in different-but-similar domains. Transferring data in this way can help reduce usage costs while also improving performance. Despite this benefit, this type of learning only works well if the original and target tasks are from the same domain [9].

There has been growing interest in unsupervised learning as a strategy for learning useful feature representations in order to avoid the complexity of manually annotating a dataset. Using unsupervised learning, a system could learn rich features using unlabeled data. Currently, there are two ways to learn feature representation via unsupervised learning: generative or SSL. In the former, latent embedding, in the form of image representations, is used for learning feature representations. These approaches typically utilize auto-encoders [10,11], adversarial learning [12,13], or jointly modelled representations and data [14] to achieve this. The downside of generative approaches is that they work in pixel space—this is expensive in terms of resources as it necessitates in-depth detail, which may not be needed for the actual process of representation learning [15].

In addressing these challenges, self-supervised learning, or SSL, has emerged to utilize unlabeled data for training, as it eliminates manual annotation as a requirement for learning representations [16]. SSL is particularly popular in the computer vision and natural language-processing fields. Research has demonstrated that self-supervised representations can compete with their supervised counterparts. In SSL, a feature extractor completes a pretext task on an unlabeled dataset. This extractor then computes generic representations for other downstream tasks, such as object classification and detection. Recent research in this area has found similar accuracy levels between SSL and supervised classifications [17,18], especially when the size of the labeled training set is small. SSL has attracted researchers for its data efficiency and improvements in model generalization.

There are two types of SSL: auxiliary pretext tasks and contrastive learning [19]. Early methods of SSL primarily defined auxiliary pretext tasks as a way to learn representations using pseudo-labels, or labels that were created automatically based on the dataset’s attributes. These were then used for tasks such as classification, detection, and segmentation, among others. Auxiliary pretext tasks can include predicting the rotation degree [20], filling in a missing part of an image [21], colorizing a grayscale image [22,23,24], predicting the relative position of a patch [25], and more.

Contrastive learning is a discriminative model that currently achieves state-of-the-art performance in SSL [15,18,26,27]. Unlike auxiliary pretext tasks, which learn using pseudo-labels, contrastive learning uses positive or negative image pairs to learn representations. It does this by discriminating between augmented views of images. For example, in one image, the representations may appear to be close, while in another the representations appear far away; noting this difference in perspective helps the model learn a useful representation. Contrastive learning has proven its usefulness in data augmentation [28], contrastive losses [26,29], momentum encoders [18,30], memory banks [31,32], and via the number of negative samples [32]. It is worth noting that recent SSL frameworks are heavily based on contrastive learning [28].

This paper surveys self-supervised feature-learning methods drawn from images. It details the motivation for this research and the terminologies of the field, and also provides an examination of pretext tasks and SSL methods, as well as contrastive learning. It further examines state-of-the-art SSL methods and compares these results to those obtained by supervised learning. Finally, this paper discusses both further considerations and ongoing challenges faced by SSL.

The reminder of the survey is structured as follows: Section 2 describes feature representation learning schemes. Section 3 introduces the pipeline and motivation of auxiliary pretext learning, while Section 4 describes the framework of contrastive learning. Section 5 and Section 6 outline the different categories of SSL and review recent research on these techniques. Section 7 compares the performance of different SSL methods on multiple datasets and downstream tasks. Section 8 explores potential challenges and possible future directions. Section 9 concludes the survey.

## 2. Feature Representation Learning Schemes

### 2.1. Transfer Learning

**Figure 1:**
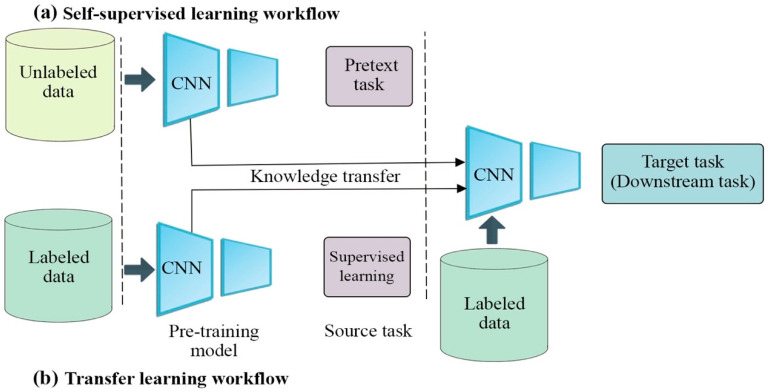
The workflows of SSL and TL. The workflows of SSL and TL are similar, with only slight differences. The key difference between TL and SSL is that TL pre-trains on labeled data, whereas SSL utilizes unlabeled to learn features, as shown in the first step. In the second step, SSL and TL are the same: both techniques are further trained on the target task, but we need only a small number of labelled examples.

Transfer learning (TL) has been presented as an effective solution for constructing robust feature representations when the training set for a given problem is small. As its name suggests, TL aims to transfer knowledge and learned features from one task (the source task) to another related target task, just as a person can utilize the same knowledge across different projects. To do this, TL trains the model on a large labeled dataset and then treats this model as a starting point in the target task’s training, without learning from scratch. This dataset creates the target task’s representation model, using the same architecture as the source task. The initialized representation network in the target task is then further trained on the target dataset. TL workflow is presented in Figure 1b.

It has been demonstrated empirically and theoretically that TL provides performance improvements and also enhances generalization in target tasks. Several deep learning models—such as AlexNet [33], VGG16 [1], Inception [2], and ResNet [2]—are publicly available as pre-trained models for transfer learning; these models have been trained on an ImageNet [34] dataset containing 1.2M high-resolution images belonging to 1000 classes. Thus, previously obtained knowledge is transferred to new tasks. This process is the foundation of TL in computer vision [35].

Despite these successes, one drawback to TL is its utilization of labels to learn network weights. While these labels may be accurate in the source task, they may not generalize well in the target task [36].

### 2.2. Unsupervised Learning: Generative Modeling

Often, unsupervised learning utilizes reconstruction. The most promising are autoencoders [10,37] and generative adversarial nets (GANs) [12]. Autoencoders use an encoder network to create feature representations with appropriate annotations, so that they can be reconstructed by a paired decoder. Variants, such as those described in [10,11], include variational auto-encoders and denoising autoencoders, but each of these use a similar model in which input data is reconstructed as an output, thereby earning them the name “auto-encoded data”, or AED. A successful feature representation, then, should include the data needed to recreate its input.

GANs, on the other hand, use adversarially trained generators and discriminators to learn feature representations via input noises, which are essentially the feature representations of the output. These input noises contain the data needed to produce the corresponding image. To create these feature representations, an auto-encoder architecture trains an encoder and then uses the generator as the decoder, which allows the encoder to produce the original image through the generator [14,38]. This method captures the best of both AED and GAN systems, leading to improved feature representations and increased popularity in both supervised and semi-supervised tasks [39].

### 2.3. Self-Supervised Learning

Recently, SSL has emerged as another popular approach. SSL is different from other techniques because it does not require manual labels. SSL is a hybrid approach, meaning that, in the pre-training fine-tuning stage, it utilizes both supervised and unsupervised learning [30]. To do this, SSL generates a supervisory signal from unlabeled data that it then uses to learn representations when annotated data is unavailable [40]. This eliminates the need for annotated data. Supervised learning in SSL can be used to train models using annotations created directly from the raw data itself [41].

SSL can be separated into two task types, pretext and downstream. The former learns representations through supervised learning, generating labels from the data itself. Once this learning is complete, the model takes learned representation from the pretext task and fine-tunes them to the downstream task [42]. Figure 1a delineates SSL’s workflow approach [41]. SSL can be further classified into two types of learning: auxiliary pretext tasks learning and contrastive learning.

Figure 1 shows the difference between TL and SSL workflows. Both TL and SSL consist of two steps: pre-training on a source task and then fine-tuning on the target/downstream task. The pre-trained weights are used to initialize the weights of the model in the target task, where the architecture of the source and target tasks are the same. The key difference between TL and SSL is that TL pre-trains on labeled data, whereas SSL utilizes unlabeled data to learn features.

In the field of SSL, convolutional neural networks (CNNs) [43] and ResNet are widely used as the backbone through which most SSL methods learn representations. CNNs consist of a stack of convolutional and pooling layers followed by a fully connected layer; the output is a softmax classifier. CNNs are often used in image recognition, object detection, recommender systems, and sentiment analysis. They can also detect features without human supervision, which makes them popular among researchers. ResNet [2] is often used in conjunction with CNN frameworks. Originally proposed by He et al., ResNet sends the feature map to the next convolution. Following that, the CNN extracts image representations from each layer to compile and recognize this image. This strategy is particularly effective to learn features via contrastive learning.

### 2.4. Contrastive Learning

Contrastive learning has shown great success in unsupervised learning. It pulls samples with the same class (a positive pair) close to each other, while driving diverse samples (or negative pairs) apart in the latent embedding space through contrastive loss. In doing this, contrastive loss minimizes the latent embedding distance between positive pairs while also simultaneously maximizing the distance between negative pairs [44]. Contrastive learning, as a concept, stems from human learning patterns, because humans can identify objects without remembering every small detail about the object. Important elements in this type of learning include: a large batch size for negative data, accurate data augmentation, and a learnable, nonlinear transformation between the contrastive loss and the representation [45,46]. Contrastive learning has demonstrated great performance in both computer vision and natural language-processing applications.

To achieve that performance, contrastive learning focuses on the similarities between different views of the same image. These images may be learned either directly or indirectly, or through instance discrimination or cluster prototypes, respectively. Instance discrimination compares pairs of images to identify which are most similar; it then moves those together while pushing dissimilar images apart [47]. To do this, SSL contrastive approaches utilize contrastive loss, encoder networks, and data-augmentation methods.

Learning representations by contrastive learning can be divided into different classes: instance discrimination, clustering-based discrimination, momentum contrast, and contrastive learning with only positive samples.

## 3. Auxiliary Pretext Task Learning Frameworks

Auxiliary pretext tasks typically operate as follows.

### 3.1. Unlabeled Data

The main goal of SSL is to avoid costly and time-consuming data annotations. Performing SSL, then, first requires the collection of unlabeled data. This step is easy, since the world is full of unlabeled, free data, such as images, videos, and texts.

### 3.2. Pretext Tasks

In the second step, SSL learns representations through pretext tasks. In auxiliary pretext methods, the model learns automatically by obtaining supervision signals directly from the data, without manual annotation. An enhanced objective function assists with this, teaching the model robust feature representations, which are needed to solve downstream tasks such as object detection and classification [48,49].

**Figure 2:**
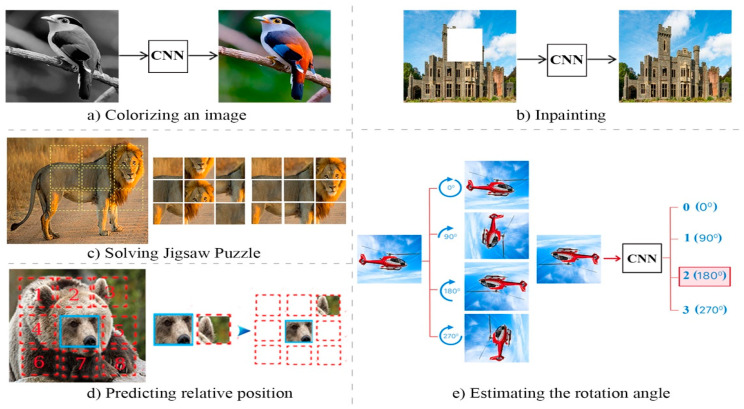
Several examples of pretext tasks. Pretext tasks easily generate pseudo-labels from the data (images) itself. Solving pretext tasks allows the model to extract useful latent representations that later improve the downstream tasks.

Designing an appropriate pretext task requires domain-specific knowledge. This is a key element of SSL, as pretext tasks can be designed for any data type, including audio, text, image, and video [48]. As shown in Figure 2, this can range from grayscale images [23], predicting a missing pixel [21], exemplar-based methods [12], rotation [20], and patch context and jigsaw puzzles [25,50], among others. Models using these different pretext tasks have, then, accomplished higher performance on various downstream tasks, a success which has been linked to pretext task design. The drawback of this method is that while good representations do emerge, they are handcrafted and may, therefore, lose their generalizability [41].

The pretext tasks in contrastive learning are data augmentations. This helps the model learn invariant representations by using distorted images to learn similarities between the representations.

### 3.3. Downstream Tasks

Pretext tasks allow the model to learn useful feature representations or model weights that can then be utilized in downstream tasks. These tasks apply pretext task knowledge, and are application-specific. In computer vision, they include image classification, object detection, image segmentation, pose estimation, etc. [48,49]. Learned feature representations and model weights should be evaluated to ensure quality. This can be accomplished in one of two ways, through either fine-tuning or using a linear classifier. To fine-tune, the model weights obtained from the pretext task are used as an initialization for which to train a new model, thereby updating all weights. To use a linear classifier, a small, labeled dataset is trained via a pretext task to perform a downstream task, freezing the weights of the rest of the model [51]. The downstream task still requires a labelled dataset, but only a small one, to achieve a good performance. If we train the deep learning model using a small number of labeled examples, and try to solve the downstream task without this pretext task, the model will produce a low accuracy.

## 4. Contrastive Learning Framework

### 4.1. Data Augmentation

The purpose of data augmentation in contrastive learning is different than in supervised learning tasks. Data augmentation aims to map an image into different views via stochastic transformations [52], which can be considered a handcrafted pretext task. The most important components for the success of contrastive learning are data-augmentation methods, as evaluated in [26], as many of the highest-performing contrastive methods use data augmentation.

**Figure 3:**
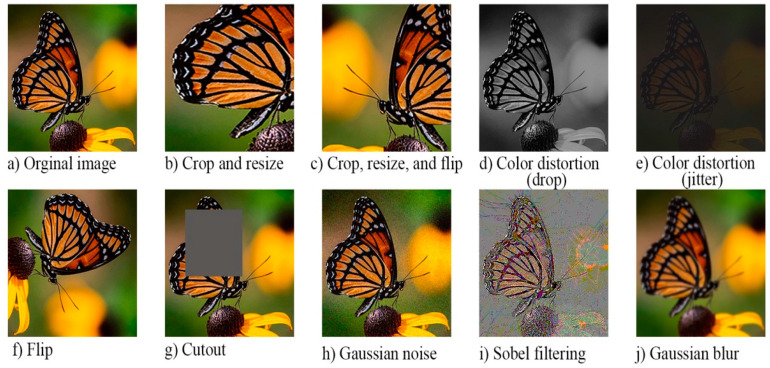
Different methods of data augmentation. It is common practice to combine multiple types of data augmentation (e.g., cropping, resizing, and flipping) for higher-quality learning and better latent features.

The data-augmentation technique T(.) maps the given input image x into different views, such as positive pairs $x_{1}^{+}$ and $x_{2}^{+}$. Doing this changes the image’s visual appearance without altering its semantic meaning. Heavily augmented data is key, because it models the complicated nature of identifying data without labels. In order for data augmentation to succeed, it must challenge the model. If, for example, the model pairs images too quickly, it has not learned, and the augmentations may be too simple [52,53]. There are several examples of augmentation, such as random cropping and resizing, random flipping, translation, color distortion, Gaussian blurring, color jitters, and multi-crop augmentation, etc., as shown in Figure 3. The research in [26] has shown that combining multiple data-augmentation techniques yields an effective representation [49].

### 4.2. Encoder f(.)

The encoder f (. ) aims to extract feature representations. To do this, it uses two augmented images, $x_{1}^{+}$ and $x_{2}^{+}$, and then extracts embedding vectors $h_{i}$ and $h_{j}$. ResNet-50 is often used as the encoder, and its variants are typically chosen as the CNN encoder. The more layers that a network has, the richer the extractions. These deeper networks contain more features, which themselves contain more semantic information than those obtained from shallower networks [26,54]. Contrastive learning is a pre-training technique in which an image encoder operates as a feature extractor in downstream tasks [55]. There are three major types of encoders in contrastive learning, as proposed in [30]: image encoders (denoted as ℎ), momentum encoders (denoted as $h_{m}$), and dictionaries (denoted as $h_{d}$). The first uses either an input or an augmented input and outputs a feature vector. Similarly, the second also outputs a vector, this time called a key vector, but the difference is that the architecture is updated more slowly than the image encoder. Finally, the dictionary method queues key vectors from the momentum encoder. Unlike the other two methods, the dictionary updates dynamically during pre-training [55].

### 4.3. Base Header g(.)

After extraction, embedding vectors $h_{i}$ and $h_{j}$ pass through a multilayer perceptron (MPL) called g(h). This base header *g*(.) maps and then passes the representations to a contrastive loss function [56]. Though this is an extra step, it has been proven to help achieve better results. Recently, refs. [18,26,57] have all shown that adding an MLP alone can increase the learned representations’ quality. On ImageNet, it improved top-1 classification accuracy by more than 10% in [26], and by 5.6% in [18].

### 4.4. Contrastive Loss

Contrastive loss functions minimize the latent embedding distance between positive pairs while also simultaneously maximizing the distance between negative pairs. Different functions have been utilized for contrastive learning, including NCE loss [58], InfoNCE loss [59], and NT-Xent loss [60]. Typically, these employ the noise-contrastive elimination (NCE) method to learn datasets. This method helps the model pull similar images together and push dissimilar ones apart. To do this, NCE uses nonlinear logistic regression, as described in [59]; this helps the model identify data from artificially generated noise. SimCLR [26] is then used to normalize loss (NT-Xent) and find positive pairs [60]. Given a mini-batch of unlabeled samples ($x_{1},\;x_{2},\;\ldots ,\;x_{N}$), stochastic augmentation T(.) is implemented to generate two different views, $x_{i}^{+}$ and $x_{j}^{+}$, of the given sample x; the different views are then fed through encoder f(.) to obtain the embedding vector extract, denoted as $\left(z_{i},\;z^{+}\right)$, as a positive pair extracted from base header g(.). This is expressed mathematically as follows:

$${ℒ}_{NCE}=-log\;\frac{exp\left(sim\left(z_{i},z_{j}\right)/\;\tau \right))}{\sum_{k=1}^{2N}I_{\left[k\neq i\right]}exp\left(sim\left(z_{i},z_{k}\right)/\;\tau \right)}$$

where $I_{\left[k\neq i\right]}\in \left[0,\;1\right]$ is an indicator function equal to 1, if k≠i and τ are temperature hyperparameters (which assist in regulating penalties on hard-negative samples [61]), and where N is the number of examples in which, for each example, two augmented views (positive pair $x_{1}^{+}\;\mathrm{and}\;\;x_{2}^{+}$) are generated from each given example x. The total number of augmented pairs is 2N, and there are 2(N−1) negative augmented examples from other examples in the dataset.

The $sim\left(z_{i},z_{j}\right)$ is a function that measures the similarity between embedding representations $z_{i}$ and $z_{j}$. Generally, a cosine function is the most common, defined as follows:

$$sim\left(z_{i},z_{j}\right)=\frac{z_{i}.z_{j}}{∥z_{i}∥.∥z_{j}∥}$$

where the notation ∥.∥ is the Euclidean norm of the vector. The cosine function measures the angle between two non-zero vectors in a d-dimensional space. At zero degrees, the cosine similarity is one. At any other angle, the cosine similarity ranges from one to negative one.

InfoNCE [59] is widely applied as contrastive loss when training contrastive models. In these instances, InfoNCE is commonly used for contrastive loss because it corresponds to cross-entropy loss, which estimates the information shared by a pair of images. It discriminates a positive pair $\left(z_{i},\;z^{+}\right)$ from its related *k* negative pairs, written as $\left(z_{i},z_{1}^{-}),\;(z_{i},z_{2}^{-}),\;\ldots ,\;(z_{i},z_{k}^{-}\right)$ [62]. The InfoNCE formula is defined as follows:

$${ℒ}_{infoNCE}=-log\frac{exp\left(sim\left(z_{i},\;z^{+}\right)/\;\tau \right)}{exp\left(sim\left(z_{i},\;z^{+}\right)/\;\tau \right)+\sum_{j=1}^{k}exp(sim(z_{i},z_{j}^{-})/\;\tau )}$$

When the labels are clean and an appropriate number of negative samples are used, the lower bound of the mutual information estimate is higher. This typically generates better performance [62]. The formula for calculating this lower bound between $z_{i}$ and $z^{+}$ is as follows:

$$I\left(z_{i},\;z^{+}\right)\geq log\left(k+1\right)-{ℒ}_{infoNCE}$$

## 5. Approaches to Auxiliary Pretext Tasks

### 5.1. Context Prediction

**Figure 4:**
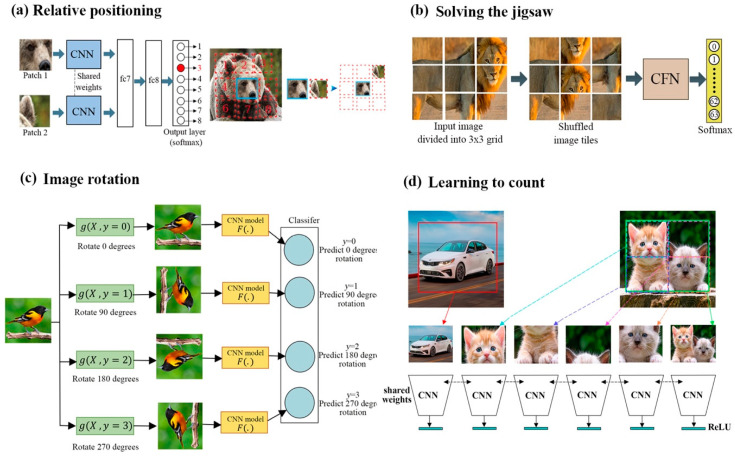
Different models of context prediction. (**a**) A pair of patches extracted randomly from each image train the CNN to identify a neighboring patch’s position in contrast to the initial patch. The weights between both CNNs are shared. (**b**) Learning representation by solving jigsaw puzzles with 3 × 3 patches. (**a**) is the original image; (**b**) is the puzzle created by shuffling the tiles using a pre-defined permutation; (**c**) is the feeding of shuffled patches into a CNF network trained to recognize permutations. (**c**) An illustration of SSL using the rotation of an input image. The model learns to predict the correct rotation from four possible angles (0, 90, 180, or 270 degrees). (**d**) proposes object counting as a pretext task for learning feature representation, thereby training a CNN architecture to count.

Context prediction is a class of SSL in which the model can predict the approximate position of image patches; to be able to do this, the model must learn spatial context to understand where these image patches belong. For example, the model developed by Doersch et al. [25] predicts the relative position between the central patch and a second patch selected randomly from its eight neighboring locations; these are numbered sequentially from 1 to 8, as depicted in Figure 4a. Each patch is fed into a convolutional neural network that follows the AlexNet architecture, where weights are shared between corresponding layers in both architectures, which then fuse into a fully connected layer. The final layer is a softmax that can predict the probability of each spatial configuration. In order to avoid overfitting, a gap was added between patches; this jittered each patch location randomly by up to seven pixels, which scaled down some images and dropped color channels, which, therefore, prevented chromatic aberrations.

Noroozi et al. [50] expanded this idea to tackle more complicated issues. They proposed a context-free network (CFN) to solve jigsaw puzzles with 3 × 3 patches by estimating which transformations were used when reordering the puzzles. Each patch passed through a Siamese convolutional layer independently, as shown in Figure 4b. Then, the features were concatenated into a fully connected layer. The output estimates the index of the chosen permutation from 64 classes, with these classes chosen from 9 permutations. The learned features were then transferred to object detection and recognition tasks, and their results beat unsupervised methods. Gidaris et al. [20] rotated an input image randomly by one of four angles (0, 90, 180, or 270 degrees), and they trained CNN on a four-class classification problem to predict the correct rotation (see Figure 4c). The authors found that the training was significantly improved by feeding four rotated images into the CNN simultaneously, instead of a single, randomly rotated image. Learning the rotation angle in this manner allowed the model to learn objects such as eyes, noses, and heads. The learned sematic features have proven useful in object detection, segmentation, and image classification.

Noroozi et al. [63] employed object counting as a pretext task for improving learned representation through scaling and tiling. The former takes advantage of the fact that visual primitives are invariant to scaling and rotation, while the latter exploits the fact that the number of visual primitives in each tile should equal the number in the entire image. By doing this, models enforce the notion that the counting feature between any pair of randomly chosen images will always be different; this lessens contrastive loss and allows the model to learn representations successfully.

### 5.2. Colorization

**Figure 5:**
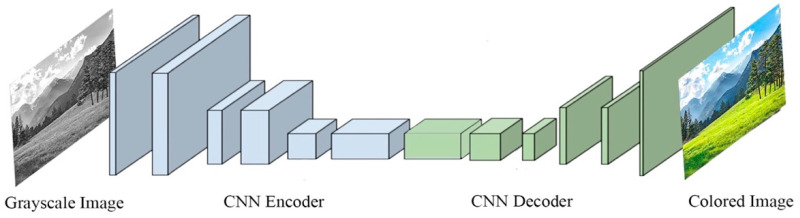
Colorization as a pretext task for learning representation. The CNN is trained to produce real-color images from a grayscale input image.

In SSL, colorization is a helpful pretext task that functions as a cross-channel encoder. The trained model restores full information about the color to each pixel. Many works based on colorization have been proposed [22,23,24]. For example, Zhang et al. [26] proposed a CNN that learns to colorize grayscale images as a pretext task. As shown in Figure 5, the architecture of this proposed CNN is similar to an autoencoder, though it uses separate image channels for both the input and the output. The experimental results have shown that learning feature representations via colorization as a pretext task is effective for solving object detection and segmentation problems; it is also more useful in image classification as compared to other self-supervised methods. Further image manipulation was suggested by Larsson et al. [24], whose ColorProxy employed a VGG-16 [1] network architecture that was pre-trained on ImageNet and fine-tined for colorization; their model could extract, from each convolutional layer, a hyper-column descriptor from each individual pixel. This allowed Larsson et al.’s model to successfully manipulate images further. Learned feature representation via colorization allows users to obtain state-of-the-art results on PASCAL VOC semantic segmentation while improving performance from 50.2% to 60.0% mIU.

### 5.3. Generative Modelling

**Figure 6:**
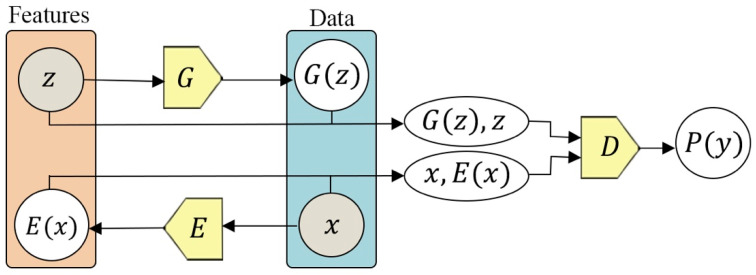
The architecture of a BiGAN. Using this technique, both (z and E(x)) and (G(z) and x) have the same dimensions. The concatenated pairs [G(z), z]  and [x, E(x)] are the two inputs of the discriminator D. Both the generator G and encoder E are optimized using the loss created by the discriminator D.

Donahue et al. [14] proposed an extension of the generative adversarial network (GAN) called Bidirectional Generative Adversarial Networks (BiGAN). Its architecture is illustrated in Figure 6. BiGAN encoders can learn feature representation for visual tasks. To do this, the authors of [12] included the generator G from a GAN as well as additional encoder neural networks (E) that map the data (x) to latent representation (z), while the generator maps the arbitrary latent distribution (z). to data (x), similar to a standard generator in GANs. The discriminator in BiGAN discriminates between the joint space of input data and latent presentation (x, E(x)) versus (G(z),z) from the generator (G), where the latent representation is either an encoder output E(z) or a generator input z. Their results show learning feature representations improves auxiliary supervised discrimination tasks, as their results are competitive with other approaches.

**Figure 7:**
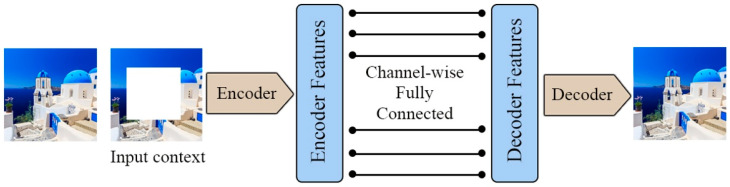
The architecture of the context encoder. It is a simple encoder–decoder pipeline.

Pathak et al. [21] presented context encoders to generate missing regions within an image. To do this, the image with missing regions is inserted into the context encoders as an input; the output is the missing pixels. The overall architecture of context encoders is depicted in Figure 7, a straightforward encoder-to-decoder pathway. Using the input with missing pixels, the encoder creates a latent feature representation; the decoder then uses this representation to produce the missing pixels. In order for this to be effective, the context encoder needs to understand the content of the image and then create a reasonable prediction of the missing pixels. The model is taught to do this through a combination of reconstruction $\left(L_{2}\right)$ loss and adversarial loss. Reconstruction $\left(L_{2}\right)$ loss aims to capture the overall structure of the missing content and context, while adversarial loss works similar to GANs, which predict realistic, missing-image content by choosing one particular mode from the distribution. In addition to producing semantic inpainting tasks, the context encoder is a powerful technique for learning feature representations for CNNs pre-training on classification, detection, and segmentation tasks.

**Figure 8:**
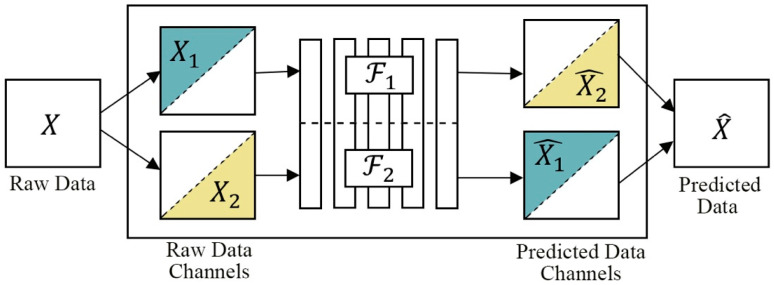
A split-brain autoencoder architecture. Comprised of two sub-networks, *F*_1_ and *F*_2_, the model is trained to predict data using the other network’s hypothesis to complement its own. Combining both hypotheses predicts the full image.

Zhang et al. [37] proposed a split-brain autoencoder that consists of two disjointed sub-networks trained as cross-channel encoders (see Figure 8). Each sub-network aims to produce a data subset. The first network $\left({ℱ}_{1}\right)$ is trained to predict $X_{2}$ from $X_{1}$ ($\hat{X_{2}}={ℱ}_{1}\left(X_{1}\right)$), and another network $\left({ℱ}_{2}\right)$ predicts $X_{1}$ from $X_{2}$ ($\hat{\;X_{1}}={ℱ}_{2}\left(X_{2}\right)$), as shown in Figure 8. This means that the two sub-networks ${ℱ}_{1}$ and ${ℱ}_{2}$ are trained to hypothesize in a way that complements the other. This difficult task allows the model to learn high-level abstractions or semantics in comparison to traditional autoencoders and other SSL methods.

### 5.4. Future Prediction

Oord et al. [59] proposed a model called contrastive predictive coding (CPC) to learn effective representations from any type of data presented as an ordered sequence, including speech, text, video, and even images, viewed as a sequence of pixels. CPC generates a rich representation by predicting future samples in the latent embedding space, using powerful autoregressive models. Contrastive loss is employed to incentivize the latent embedding space to capture information that then maximizes the mutual information between the current and future samples.

### 5.5. Clustering as Pretext Task

**Figure 9:**
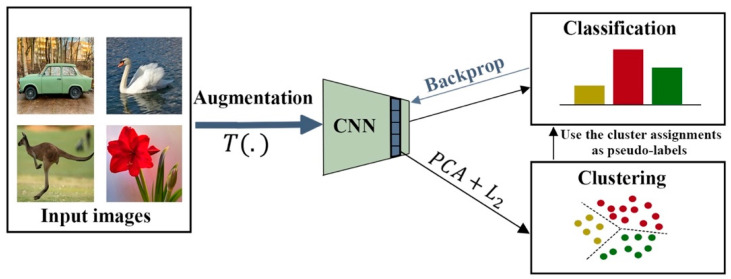
An illustration of the DeepCluster pipeline for learning representations.

Clustering can also be employed for learning deep feature representation. For example, Caron et al. [17] used k-means assignments to learn feature representations. The general structure of DeepCluster, as shown in Figure 9, is a large-scale, end-to-end learning method that iteratively clusters the features of images into groups using the k-means algorithm. Cluster assignments are then used as “pseudo-labels” to optimize the weights of the CNN by predicting cluster assignments.

## 6. Contrastive Learning Methods for Learning Visual Representation

### 6.1. Instance Discrimination

Many recent works on contrastive learning have studied instance discrimination [18,26,30], an approach that classifies each image separately and then uses data augmentations to train the model. Top-performing contrastive learning methods, such as SimCLR [26] and MoCo [30], utilize instance discrimination as a pretext task, which has been demonstrated to outperform its supervised counterparts on downstream tasks. Instance discrimination methods train the network so that two augmented versions of a sample should have comparable representations. Likewise, two augmented versions of two different images should have incomparable representations. Formally, this approach generates two augmented images (a positive pair) $x_{1}^{+}$ and $x_{2}^{+}$ from any given x. Therefore, for N images in the batch, 2N augmented views are generated. Pairing each image $x_{i}$ in the batch with all other images (indexed j) will maximize the number of negative images. This generates 2(N−1) negative augmented images from other images in the dataset. The positive and negative images then pass through an encoder to obtain latent representation. Afterwards, contrastive loss is implemented to improve the likeness in positive pairs and unlikeness in negative pairs [59,64].

When using instance discrimination, self-supervised methods utilize two key elements: one is contrastive loss [59], and the second is data augmentation [28]. The former compares image features directly, while the latter delineates features’ invariances.

### 6.2. SimCLR

**Figure 10:**
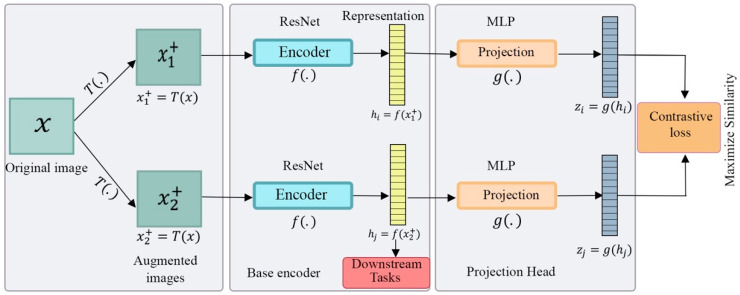
The structure of SimCLR. Data augmentation T(.) is applied on the input image x  to generate two augmented images $x_{1}^{+}$ and $x_{2}^{+}$. A base encoder network f(.)  and a projection head g(.) are trained to maximize the similarity between the augmented images using contrastive loss. After completing the training process, the representation h is used for downstream tasks.

Chen et al. [26] proposed SimCLR, a framework for learning useful presentations based on contrastive learning by maximizing the similarity between the original data image and different, augmented views of it. This method also minimizes connections between altered views of different images in latent space using contrastive loss. As shown in Figure 10, the SimCLR architecture contains a base encoder using a ResNet architecture that produces an embedding representation from augmented images $h_{i}=f\left(x_{i}^{+}\right)=ResNet\left(x_{i}^{+}\right)$. The output is taken after the average pooling layer. Then, the projection head is built using MLP, with one hidden layer containing an ReLU activation function, where contrastive loss is applied to take the embeddings $h_{i}$ and produce latent space $z_{i}=g\left(h_{i}\right)$. When the parameters are updated in this way, comparable representations attract each other, and incomparable ones are repelled. Research has shown that increasing the architecture’s depth and width, batch size, and epochs makes contrastive loss extraction better at achieving high performance. Increasing the batch size specifically is beneficial for SimCLR to ensure the presence of enough negative pairs. For data augmentation, the accuracy was highest when both random cropping and random color distortion were applied after examining the composition of each data-augmentation operator. The downside, however, is that while a large batch size has improved the performance, it also leads to higher computational costs.

The same team released an improved version of SimCLR based on semi-supervised learning, called SimCLRv2 [29], that involves self-supervised pre-training (in a task-agnostic way), followed by supervised fine-tuning in which only 1% or 10% of the labeled images were available. They demonstrated that using fewer labels improved accuracy. In addition, re-labelling the data allowed task-specific predictions to be transferred into a smaller network. Doing this can improve smaller ResNet-50 networks while maintaining accuracy, as the negative pairs in SimCLR can be used as the positive pairs.

According to [65], instance discrimination leads to class collision problems. This means that instance similarities will need to be pushed apart, which can hurt representation quality [65]. Identifying and using these similar instances, then, is important for achieving high performance in learned representations.

### 6.3. Memory Bank

Recent work has shown that generating a robust visual feature representation requires comparing the current sample with a large number of negative samples in contrastive loss. In a mini-batch stochastic gradient descent optimizer, however, including an appropriate number of negative samples may overly increase the mini-batch size, which can create resource challenges. To address this, a memory bank has been proposed as an effective technique for storing the feature representations of large negative samples without increasing the size of the mini-batch. Using this technique, the memory bank ℳ is comprised of a feature representation $m_{I}$ for each sample I. The representation $m_{I}$ is key to this method, as it is an exponential moving average of feature representations $f\left(v_{I}\right)$, all of which were previously calculated. Negative samples such as $f\left(v_{I}^{'}\right)$ can then be exchanged for their memory bank representations, $m_{I^{\prime }}$; doing this does not increase the size of the training batch, which eliminates the need for additional computational resources.

One such example is PIRL [66], which uses a memory bank comprised of the moving averages of all learned representations, providing significant negative samples for training. To do this, the model must generate data representations of any images that are covariant to the pretext tasks, particularly jigsaw or rotation-degree prediction tasks. To do this, PIRL creates an image representation that is, one, similar to transformed representations, and two, different from other samples’ representations. The results obtained by PIRL outperformed supervised pretraining in object detection. Some works [18,30] further suggested the use of a momentum contrast mechanism in which the query encoder learns representations from a slow key encoder; it then maintains a memory buffer to store high quantities of negative samples.

**Figure 11:**
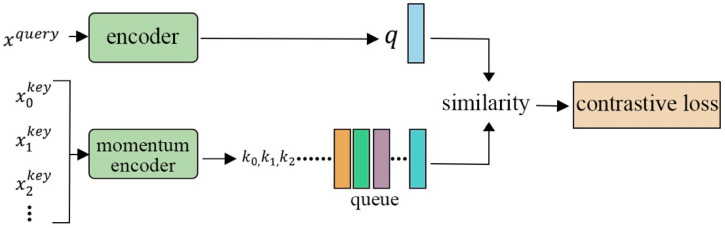
The structure of MoCo. MoCo uses two encoders, an encoder and a momentum encoder.

He et al. [30] utilized a different approach, called momentum contrast (MoCo), which uses and updates a memory bank. This method splits each image into a query, and then creates a key by performing two different augmentations. Figure 11 shows how the query and all keys are then passed through an encoder, which creates embeddings. The similarity is then calculated between query/key pairs. To update the momentum encoder, the model computes the contrastive loss and backpropagates it through the encoder. These weights are updated at every iteration for the highest accuracy. The model and encoder rely on the memory bank, but this update scheme allows it to instead pursue the exponential moving average, or momentum update. Though this was a self-supervised model, its success rate was similar to those of supervised models.

MoCo v2 [18] is an improved version of MoCo with simple modifications, including replacing a two-layer MLP head with ReLU for the unsupervised training stage and blur data augmentation. Because of these changes, MoCo has outperformed the best SimCLR, despite having fewer epochs and a smaller batch size.

### 6.4. Contrastive Learning without Negatives

**Figure 12:**
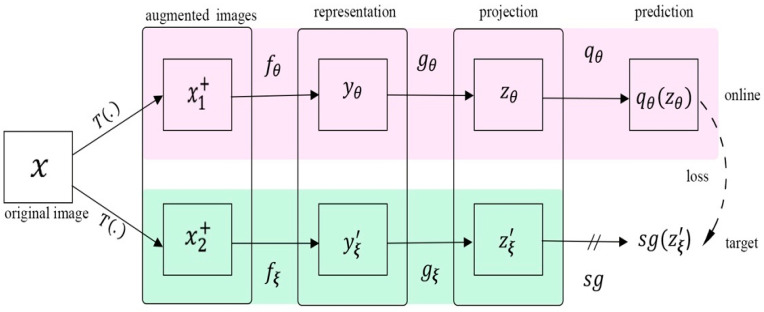
A BYOL architecture. BYOL reduces similarity loss between $q_{\theta }$($z_{\theta }$ ) and sg ($z_{\xi }^{'}$ ), where θ represents the trained weights, ξ represents an exponential moving average of θ, and sg means the stop-gradient. After training, everything but $f_{\theta }$ is discarded. $y_{\theta }$ represents the image representation.

Some recent works [15,67] have achieved remarkable results by only using positive examples. Grill et al. [15] proposed a new method called BYOL for learning feature representations without ever contrasting negative pairs. Instead of focusing on dissimilarity, this method focuses only on the similarity of samples and representations. They did this via bootstrapping, using the outputs as targets for enhanced representations. Using both target and online networks, BYOL interacts and learns from itself. The online network consists of three main components, as illustrated in Figure 12: the encoder $f_{w}$, which is Res-Net-50, the projector $g_{w}$, and the predictor $q_{w}$. The target network has same architecture as the online network but with different weights (ξ). To train the online network, the target network supplies regression targets; it uses an exponential moving average of the online weights w to define the parameters ξ. BYOL then feeds the input image x and its augmented image $x^{+}$ to the online network and target network, respectively, to extract embedding vectors from each network. The loss of BYOL is the mean squared error rather than contrastive loss, which aims to minimize the similarity distance between the embedding vectors, where w represents the trained weights and ξ represents an exponential moving average of w. The image representation corresponds to the output of the final average pooling layer of the online network encoder $f_{w}$, where a moving average of the online network updates the target network.

**Figure 13:**
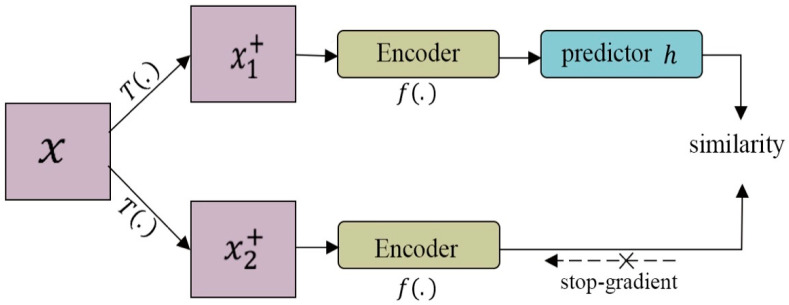
The architecture of SimSiam. Two augmented images passed through the same encoder, which is comprised of a backbone (ResNet) and a projection MLP. A prediction MLP (h) is used on one side, and a stop-gradient strategy is employed on the other to avoid collapse. The model aims to maximize the similarity between both views. SimSiam does not use negative pairs or a momentum encoder.

Other methods include Chen and He’s research [67], which demonstrated that a high-quality representation can be obtained without using either negative samples or a momentum encoder. They proposed a simple Siamese network (SimSiam) with a stop-gradient operation to learn feature presentation. SimSiam (Figure 13) takes two augmented views, $x_{1}^{+}$ and $x_{2}^{+}$, which are generated randomly from image x, and then passes each view through encoder f, which shares the weights between the two views. The encoder network is comprised of a backbone, in this case ResNet, and a projection MLP. A prediction MLP (h) is used on one side, and a stop-gradient strategy is employed on the other to avoid collapse. The SimSiam method was effective on both ImageNet and downstream projects.

### 6.5. Clustering-Based Methods

Clustering-based methods in contrastive learning [17,68,69] enforce consistency when cluster assignments are obtained from different augmented views of the same image; this is conducted instead of comparing embedding features directly, as in contrastive learning. Once this is complete, the model is trained on the cluster assignments, which are handled like pseudo-labels, similar to supervised learning.

**Figure 14:**
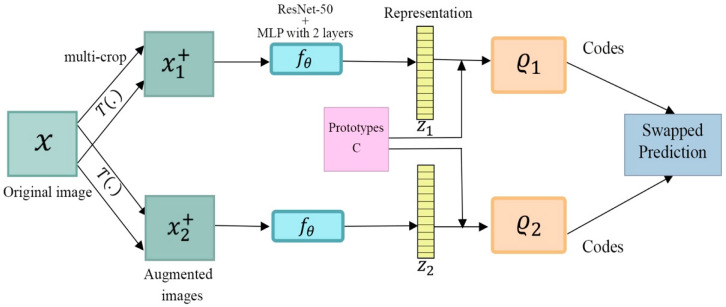
The structure of SwAV. It assigns a code to an augmented image and then anticipates that code by using a second augmentation of that image. SwAV ensures consistency by comparing features directly, the same as contrastive learning.

SwAV [17] is an online clustering method which trains features to produce reliably similar cluster assignments when given various views of the same image, as presented in Figure 14. In mining the invariant clusters of these data augmentations, the model learns rich feature representations. This is accomplished by comparing and contrasting the features of the same image, using their intermediate cluster assignments from multiple views. If the information presented is similar, then it can be used to predict feature assignments from other views. A set of K clusters is associated with a d-dimensional prototype vector vk. Each image i is transformed into two different views: $x_{i1}$ and $x_{i2}$. Each of these views is featurized with ResNet, which provides two sets of features: (f11,…, fB1) and (f12,…,fB2). Each set is then allocated to cluster prototypes using an optimal transport solver, which ensures that features are split evenly across the clusters [70]. The resulting data are then switched so that the cluster assignment yi1 of the view xi1 has to be predicted from the feature representation fi2 of the view xi2. Loss is minimized for all examples i via the following equation:

L(fi1, fi2)=l(fi1, yi2)+l(fi2, yi1).

To calculate cluster prediction loss, l(f,y) represents the cross entropy between a cluster assignment as well as the softmax of the dot products of f and all prototypes vk.

However, doing this can lead to degenerate solutions. This is prone to happen when the solutions of the k-means algorithm are all mapped to one cluster. SeLa [68] addressed this by including constraints that prevent this, using equipartitions and a simplified Sinkhorn–Knopp algorithm. PCL [69] further noted class collision and used instance discrimination and unsupervised clustering, in tandem, to address this. Though it achieves the same linear classification accuracy as MoCo v2, this method performs better on downstream tasks [65].

## 7. The Performance of Image Feature Learning

The goal of solving pretext tasks in SSL is to learn a discriminative representation that then improves downstream tasks. A common approach to evaluating the quality of learned features is either a linear classifier or fine-tuning. In both approaches, the model is trained to solve pretext tasks on a large dataset, such as ImageNet, which does not include label information. Next, a linear classifier or fine-tuner will focus on solving the pretext tasks, whose solutions will then be used to improve downstream performance. To do this, both approaches suspend the network. Finally, the image classification performance must be assessed. ImageNet [34], VOC07 [71], and Places205 [72] are common datasets for this purpose. Similarly, the Pascal VOC12 [73] dataset is often used to evaluate both object detection and semantic segmentation.

Table 1 illustrates the top-1 classification performance on different SSL methods that pre-trained on ImageNet without labels, utilized supervised linear classification on a suspended network, and were then evaluated on ImageNet, VOC07, and Place205 testing sets. ResNet is the backbone for most SSL methods. The accuracy performance is based on the number of parameters and the experimental setup. As shown in Table 1, SwAV outperforms MoCo v2 by 4.2% and closes the gaps with supervised training to less than 1%. In addition, contrastive learning methods perform better than the auxiliary pretext learning techniques on image classification.

The quality of the learned representations is evaluated by transferring them to other downstream tasks, including Pascal VOC 2007 [71] object classification and detection and VOC12 [73] instance segmentation. The model is first pre-trained on VOC07 without labels using Faster-CNN [75] and then fine-tuned on the target datasets. Then, it proceeds to the classification, detection, and segmentation tasks, which are defined as follows: classification is multi-class task, and predicts the presence of 20 object classes. Detection tasks require the locating of objects in a sample and surrounding them with a bounding box. Finally, segmentation labels each pixel in an image with its object class. As illustrated in Table 2, the results show that contrastive learning methods outperformed supervised fusion and auxiliary pretext methods. In auxiliary pretext task methods, learning features using colorization is suitable for segmentation, while predicting the context works well for detection.

**Figure 15:**
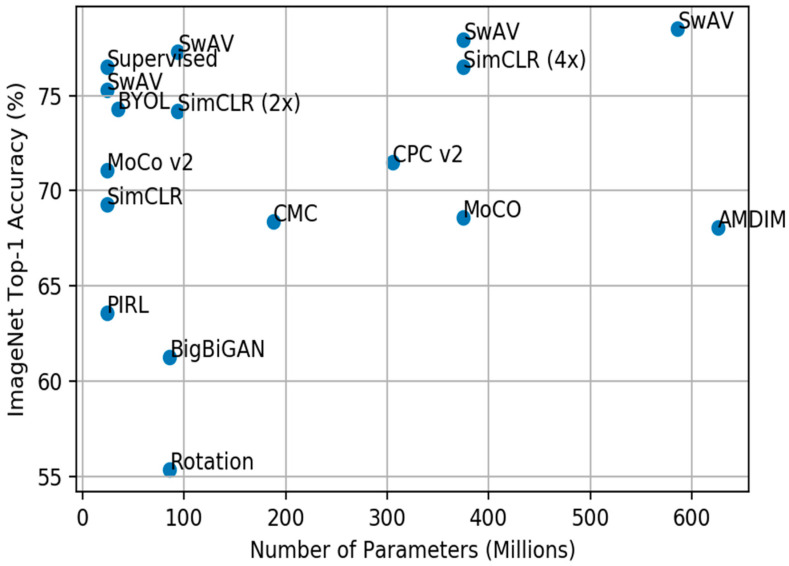
The accuracy of the ImageNet Top-1 linear classifiers, which were trained on feature representations created via self-supervised techniques with different widths of ResNet 50. All were pretrained on ImageNet.

Figure 15 shows the accuracy of the ImageNet Top-1 linear classifiers, which were trained on feature representations created via self-supervised techniques with different widths. As shown in the figure, increasing the parameters of SimCLR obtains the same performance as ResNet-50 trained on supervised learning. SwAV also beats supervised learning. It is shown empirically, then, that more complex models learn more effectively, and create better feature representations, using unlabeled data.

## 8. Discussion of Self-Supervised Learning

The existing self-supervised methods learn representations just as well as supervised methods [17,27,67,77]. However, the best SSL algorithms require training on computationally expensive devices. Even the smallest architectures, such as ResNet-50 [2], require large mini-batches, often with thousands of images, while utilizing specialized hardware [17,26], all of which is difficult in a setting with limited resources. In some cases, it may be downright impossible, as SSL does not always work on lighter models such as MobileNet-V3-Larger. To address these constraints, we must focus on networks: they should be strong and small, and they must be compatible with a system on a chip, or SoC [78].

Self-supervised models learn through pretext tasks. These tasks are not primary, but they are intended to be solved. By doing so, the model learns complicated feature representations that later assist with downstream tasks and allow for high performance, even with limited data annotations. To create the best scenario, the pretext task must be selected with care, and it must work in tandem with the model’s downstream tasks. The process of designing a proper pretext task for a given downstream task still needs more exploration. Numerous pretext tasks have been proposed, but research has not yet been conclusive. For example, in a classification scenario, rich image representations may create clusters of lower-quality embeddings, a situation in which those clusters are image classes for labelling. On the other hand, for a localization task, rich representations can create clusters of clusters, a situation in which the biggest cluster represents an approximation of an object’s location within an image [79]. Future research should focus here.

The success of contrastive learning is highly dependent on the design of its positive and negative samples. They must be designed carefully, particularly positive pairs, because data augmentations can improve representations. As demonstrated in [26,76], data augmentation has played a critical role in obtaining discriminative representations. On the other hand, in terms of negative pairs, researchers have found many ways to augment them as well. One such augmentation is InstDisc [32], which utilized a memory bank to store previous batches’ feature embeddings, a strategy that allowed for a large pool of negatives and led to greater performance. MoCo [18,30] then added a momentum encoder to further augment the data. SimCLR [26] improved its negative pairs via large-batch online training, using other pairs in the same dataset to prevent contrastive loss.

Contrastive learning methods suffer from a mode collapse problem, in which the model maps all of its input data to the same representation [80]. Different approaches have been presented to address this problem. One is the use of methods such as MoCo [26], where the loss function treats pairs of positive and negative samples differently. BYOL [15] and SimSiam [67], on the other hand, employ stop-gradient strategies as well as an extra predictor to counteract the lack of negative pairs. Refs. [17,76], furthermore, add clustering, and Barlow Twins compares two branches to reduce repetitive information [81].

## 9. Conclusions

Recent research has shown that SSL can offer high-quality visual representations without labels and, thereby, eliminate manual annotation processes. Advancements in this field have allowed researchers to obtain a better feature representation from different perspectives, which dovetails well with SSL, thanks in no small part to contrastive learning methods. This paper provided a comprehensive overview of top-performing SSL methods. It delineated the different categories of SSL and reviewed recent research into these techniques. It also presented performance analyses on the different ways to approach downstream tasks, all of which points to the importance of contrastive learning in SSL models.

## Figures and Tables

**Table 1:** Image classification with linear models on ImageNet, VOC07, and Place205 testing sets. All models are pre-trained on ImageNet without labels, using different SSL methods.

Method	Architecture	# of Param (Million)	ImageNet	VOC07	Place205
Supervised	ResNet-50	24 M	76.5	-	-
Colorization [23]	ResNet-50	24 M	39.6	-	-
Jigsaw [50]	ResNet-50	24 M	45.7	64.5	41.2
Rotation [20]	ResNet-50	24 M	48.9	63.9	41.4
NPID [32]	ResNet-50	24 M	54	-	-
BigBiGAN [74]	ResNet-50	24 M	56.6	-	-
MoCo [30]	ResNet-50	24 M	60.6	79.2	48.9
PCL [69]	ResNet-50	24 M	61.5	82.3	49.2
PIRL [66]	ResNet-50	24 M	63.6	81.1	49.8
CPC v2 [59]	ResNet-50	24 M	63.8	-	-
SimCLR [26]	ResNet-50	24 M	69.3	-	-
MoCo v2 [18]	ResNet-50	24 M	71.1	-	-
SwAV [17]	ResNet-50	24 M	75.3	88.9	56.7
**Different Architectures and Setups**					
Supervised	ResNet-50	25.6M	75.9	87.5	51.5
Colorization [23]	ResNet-50	25.6M	39.6	55.6	37.5
Rotation [20]	ResNet-50	25.6M	48.9	63.9	41.4
DeepCluster [17]	VGG16	15 M	48.9	63.9	41.4
NPID [32]	ResNet-50	25.6 M	54	-	45.5
PCL v2 [69]	ResNet-50-MLP	28 M	67.6	85.4	50.3
BYOL [15]	ResNet-50-MLP	35 M	74.3	-	-
DeepCluster [17]	AlexNet	61 M	54	-	37.5
AMDIM [57]	Custom-RN	670 M	68.1	-	55.1

**Table 2:** Evaluation of transfer learning on downstream tasks using PASCAL: object classification and detection is evaluated on PASCAL VOC 2007 [71]. Segmentation is evaluated on PASCAL VOC 2012 using Faster-RCNN. The results were obtained from [26,63,76].

Method	VOC07 Classification	VOC07 Detection	VOC12 Segmentation
Supervised [33]	79.9	56.8	48.0
Context [25]	55.3	46.0	-
Jigsaw [50]	67.6	53.2	37.6
ContextEncoder [21]	56.5	44.5	29.7
BiGAN [14]	58.6	46.2	34.9
Colorization [22]	65.9	46.9	35.6
Split-Brain [37]	67.1	46.7	36.0
ColorBroxy [24]	65.9	-	38.0
Counting [63]	67.7	51.4	36.6
PIRL [66]	81.1	80.7	-
Barlow Twins [77]	86.2	-	-
MoCo [30]	-	81.4	-
SwAV [17]	88.9	82.6	-

## Data Availability

Not Applicable.
